# Evaluating principal component analysis models for representing anatomical changes in head and neck radiotherapy

**DOI:** 10.1016/j.phro.2022.04.002

**Published:** 2022-04-13

**Authors:** Raul Argota-Perez, Jennifer Robbins, Andrew Green, Marcel van Herk, Stine Korreman, Eliana Vásquez-Osorio

**Affiliations:** a Department of Oncology, Aarhus University Hospital, Aarhus, Denmark; b The University of Manchester, Division of Cancer Sciences, Faculty of Biology, Medicine and Health, Manchester, United Kingdom; c Danish Centre for Particle Therapy, Aarhus University Hospital, Aarhus, Denmark; d Department of Clinical Medicine, Aarhus University, Aarhus, Denmark

**Keywords:** Radiotherapy, Head and neck, Anatomical deformations, Principal component analysis

## Abstract

**Background and purpose:**

Anatomical changes during radiotherapy pose a challenge to robustness of plans. Principal component analysis (PCA) is commonly used to model such changes. We propose a toolbox to evaluate how closely a given PCA model can represent actual deformations seen in the patient and highlight regions where the model struggles to capture these changes.

**Materials and methods:**

We propose to calculate a residual error map from the difference between an actual displacement vector field (DVF) and the closest DVF that the PCA model can produce. This was done by taking the inner product of the DVF with the PCA components from the model. As a global measure of error, the 90th percentile of the residual errors $({M_{\mathrm{res}}}^{90}$) across the whole scan was used. As proof of principle, we demonstrated this approach on both patient-specific cases and a population-based PCA in head and neck (H&N) cancer patients. These models were created using deformation data from deformable registrations between the planning computed tomography and cone-beam computed tomography (CBCTs), and were evaluated against DVFs from registrations of CBCTs not used to create the model.

**Results:**

For our example cases, the oropharyngeal and the nasal cavity regions showed the largest local residual error, indicating the PCA models struggle to predict deformations seen in these regions. ${M_{\mathrm{res}}}^{90}$ ranged from 0.4 mm to 6.3 mm across the different models.

**Conclusions:**

A method to quantitatively evaluate how well PCA models represent observed anatomical changes was proposed. We demonstrated our approach on H&N PCA models, but it can be applied to other sites.

## 1 Introduction

Throughout radiotherapy, geometric uncertainties such as set-up errors or anatomical changes may lead to underdosing the target or overdosing organs at risk. Margins or robust optimisation techniques can deal with these uncertainties, but their applicability is limited for complex changes. Therefore, adaptive strategies are often used for larger anatomical changes [1], [2]. However, current adaptation strategies represent a significant workload and can create bottlenecks in workflows [3], [4].

An alternative is to predict anatomical changes using mathematical models to increase plan robustness. Such models could be used to predict which patients may benefit from plan adaptation, or to identify regions where large anatomical changes are expected. Deformation models could also simulate anatomical changes during planning to increase robustness, e.g. using robust or probabilistic planning [5]. Principal component analysis (PCA) is widely used for creating such models, for instance in lung [6], [7], [8], [9], prostate [10], [11], [12], [13], [14], cervix [5] and head and neck (H&N) [15], [16]. The usefulness of such models depends on their ability to accurately simulate future changes in the patient.

In this study, we propose a toolbox to evaluate how well a given PCA model can represent anatomical changes that were not used to generate the model. These tools can be used to evaluate the model robustness, estimate global residual errors within the boundaries of the scan and highlight regions where the model struggles to capture anatomical changes. The aim of this study was to present a proof-of-principle for this method in H&N cancer using both patient-specific and population-based deformation models.

## 2 Materials and methods

PCA models can be created from a set of displacement vector fields (DVFs) to simulate anatomical changes. These DVFs use non-rigid registrations to describe the deformations between two images, e.g., the planning CT (pCT) and a cone-beam CT (CBCT).

The resulting model consists of the eigenvectors for each of the principal components of deformation, ${\overset{¯}{e}}_{i}$, the corresponding variance for each component, $v{\mathrm{ar}}_{i}$, and the mean of the input DVFs, ${\overset{¯}{v}}_{\mathrm{mean}}$. These can then be used to simulate plausible DVFs (${\overset{¯}{v}}_{\mathrm{sim}}$), following Eq. (1), where the weights, $u_{i}$, are selected from a Gaussian distribution centred at zero with variance $v{\mathrm{ar}}_{i}$.

(1) $${\overset{¯}{v}}_{\mathrm{sim}}={\overset{¯}{v}}_{\mathrm{mean}}+\sum_{i}u_{i}{\overset{¯}{e}}_{i}$$

### 2.1 Evaluation strategy for PCA-based deformation models

The proposed method presented here can be used to determine to what degree a PCA model can represent a DVF that was not used to generate the model. We refer to this DVF as the ‘reference DVF’, ${\overset{¯}{v}}_{\mathrm{ref}}$. The closest vector field to the reference DVF was generated using the model with an optimal set of weights, $w_{i}$, found using Eq. (2).

(2) $$w_{i}={\overset{¯}{e}}_{i}\cdot {\left({\overset{¯}{v}}_{\mathrm{ref}}-{\overset{¯}{v}}_{\mathrm{mean}}\right)}^{T}$$

The closest simulated DVF the model can produce was found by substituting $w_{i}$, from Eq. (2) into Eq. (1). A measure of the likelihood of this closest DVF being produced by the model, the Z-score, is presented in Supplement 1.

To quantify how close the closest DVF is to the reference DVF, we defined the residual DVF, ${\overset{¯}{v}}_{\mathrm{res}}$, as.

(3) $${\overset{¯}{v}}_{\mathrm{res}}={\overset{¯}{v}}_{\mathrm{ref}}-{\overset{¯}{v}}_{\mathrm{closest}}$$

By taking the magnitude of each vector within ${\overset{¯}{v}}_{\mathrm{res}}$, $M_{\mathrm{res}}$, we can identify local regions with larger errors. As a measure of the global model performance, we calculated the 90th percentile of $M_{\mathrm{res}}$, ${M_{\mathrm{res}}}^{90}$. The 90th percentile is commonly used in the literature [9], but the mean or a different percentile could be selected depending on the intended application.

### 2.2 Evaluation toolbox

Our evaluation scheme can be used in different ways to evaluate how well PCA-models perform. E.g., we can evaluate the robustness and stability of the model, the sensitivity of the model to random noise, and the general ability of the model to simulate anatomical changes within the patient.

**Model robustness:** To determine how robust a PCA model is to the input DVFs, a leave-one-out cross validation (LOOCV) analysis can be performed by running PCA with one of the input DVFs left out. The DVF that is left out can then be used as ${\overset{¯}{v}}_{\mathrm{ref}}$ for that iteration.

**Model sensitivity:** The sensitivity of the model to random noise and the number of components used for the evaluation can be assessed using simulated DVFs, to which Gaussian noise was added (Supplement 2).

**Model generalisability:** To evaluate whether a deformation model describes real anatomical changes, the PCA model can be evaluated against unseen DVFs from the patient.

### 2.3 PCA models examples

To test our proposed method in different settings, we created both patient-specific models (datasets 1/2) and a population-based model (dataset 3). The datasets are summarized in Table 1 and detailed in Supplement 3.

**Table 1:** Summary of patient datasets.

**Data for**	**Patient-specific PCA models**		**Population-based PCA models**	
			**Training**	**Validation**
**Dataset**	**1**	**2**	**3a**	**3b**
**Nr of patients**	24	20	20	20
**Site**	Sinonasal	Oropharyngeal	Oropharyngeal	Oropharyngeal
**Treatment technique**	All IMRT	10 IMRT, 10 VMAT	All VMAT	All VMAT
**Treatment period**	2009–2017	2008–2018	2016–2018	2016–2018
**Dose prescription**	66–68 Gy, (n = 16), 60–66 Gy (n = 8)	66–68 Gy (n = 17), 76 Gy (n = 3).	60–66 Gy	60–66 Gy
**Number of CBCTs per patient**	30–34	33–34 or 56	8–31	9–24

**Dataset 1** includes twenty-four sinonasal cancer patients collected from the DAHANCA database with 30–34 daily CBCTs per patient, under approval by the Danish Data Protection Agency (1–16-02–676-18).

**Dataset 2** includes twenty oropharyngeal cancer patients collected from a single institution with 33–56 CBCTs per patient, under internal approval (in accordance with Danish guidelines).

**Dataset 3** includes forty oropharyngeal patients collected from a single institution with 8–31 CBCTs per patients, under ethics approval from the UK North West - Haydock Research Ethics Committee, (17/NW/0060, local ref. 2018–018). Twenty patients were selected as training patients, dataset 3a. The remaining twenty patients were used for validation, dataset 3b.

**Fig. 1:**
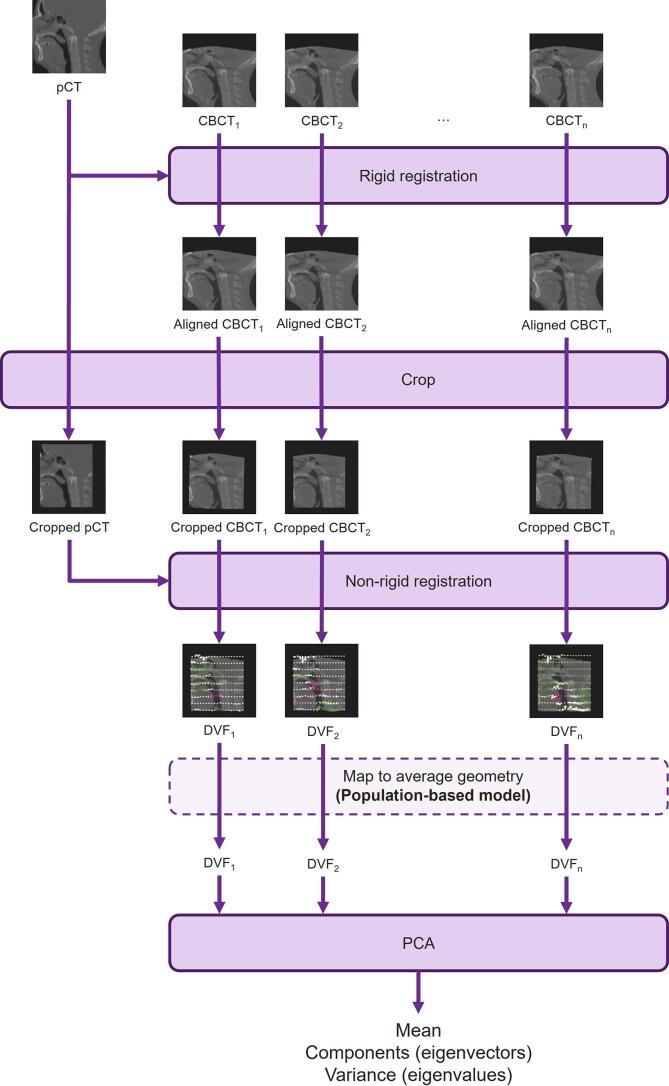
Flow chart showing the method for generating PCA models. For the patient-specific models, all CBCTs are from the same patient and are registered to the same pCT for that patient. For the population-based model, each CBCT is from a different patient and is registered to the corresponding pCT.

To generate the DVFs for PCA creation, each CBCT was first rigidly registered to the pCT based on bony anatomy, obtaining the starting point for deformation. The pCT and aligned CBCTs were then cropped to a bounding box to reduce computation time. Then, deformable registration was performed from each CBCT to the pCT, using either NiftyReg [17] (datasets 2/3) or Anaconda in RayStation [18] (dataset 1). A scheme summarising our approach is presented in Fig. 1.

For the patient-specific models, DVFs from the first 5-days of treatment were used to create each PCA model. The bounding box covered an area including the brainstem, parotids, primary CTV and the nodal CTV (if present) with a 5 cm margin in each direction excluding the shoulders. To investigate the effect of the number of input scans, models were created for two of the patients (dataset 2) using the 10 DVFs corresponding to the first two weeks of treatment.

For the population-based model, DVFs mapping a CBCT from each of the 6-weeks of treatment from each of the 20 training patients were used. To standardise the DVFs between different patients, an average patient geometry was created using a groupwise registration [19] of the pCTs of these patients. The bounding box covered an area including the brainstem, parotids, oral cavity, larynx and primary CTV with a 1.5 cm margin in each direction. All DVFs were then mapped to the average geometry using SimpleITK [20] and were used to create a single PCA model.

The authors visually inspected all deformed CTs and fine-tuned sub-optimal registrations.

We generated all PCA models using Scikit-learn in Python [21]. We report the cumulative variance for each component for the PCA models in Supplement 4.

### 2.4 Model evaluation

For all models, we evaluated robustness with the LOOCV method. We ran an investigation into the model sensitivity for the population-based model and one of the patient-specific models (Supplement 2).

As test data for the model generalisability method, we used the DVFs from the first CBCT of each of the subsequent five-weeks of treatment for patient-specific cases. For the population-based case, we used all remaining DVFs of the training patients (dataset 3a) and DVFs from all CBCTs of the validation patients (dataset 3b). In each case, we created heat maps of $M_{\mathrm{res}}$ to evaluate the local model quality and ${M_{\mathrm{res}}}^{90}$ to evaluate the global model quality.

A pass/fail threshold for ${M_{\mathrm{res}}}^{90}$ can be set depending on the specific application. As an example, we selected 4 mm.

We used all components available for evaluating the PCA models, but in practice one could restrict the number of components, e.g. many PCA models only include components that cover the largest 90% of the total variance of the model.

## 3 Results

### 3.1 Model robustness (LOOCV)

**Fig. 2:**
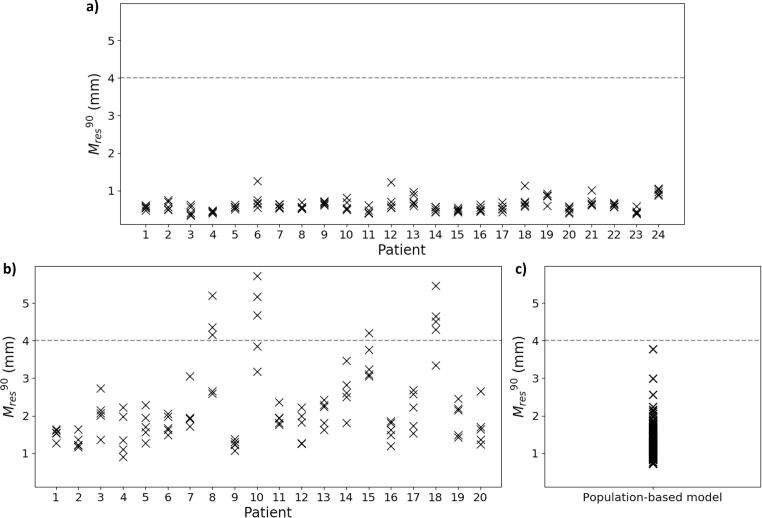
${M_{\mathrm{res}}}^{90}$ for the LOOCV for each of patient-specific model in a) dataset 1 and b) dataset 2, and for c) the population-based model (dataset 3a).

Fig. 2 shows ${M_{\mathrm{res}}}^{90}$ for the LOOCV for all models. For the patient-specific study, all ${M_{\mathrm{res}}}^{90}$ were well below the 4 mm threshold for dataset 1, while for dataset 2, models for 4 out of 20 patients had ${M_{\mathrm{res}}}^{90}$ > 4 mm. In the population-based study (dataset 3a), all ${M_{\mathrm{res}}}^{90}$ were below 4 mm.

The largest $M_{\mathrm{res}}$ were observed in the regions around the oropharynx for all datasets. Additionally, for dataset 1, the region around the nasal cavities had high $M_{\mathrm{res}}$.

### 3.2 Model generalisability

**Fig. 3:**
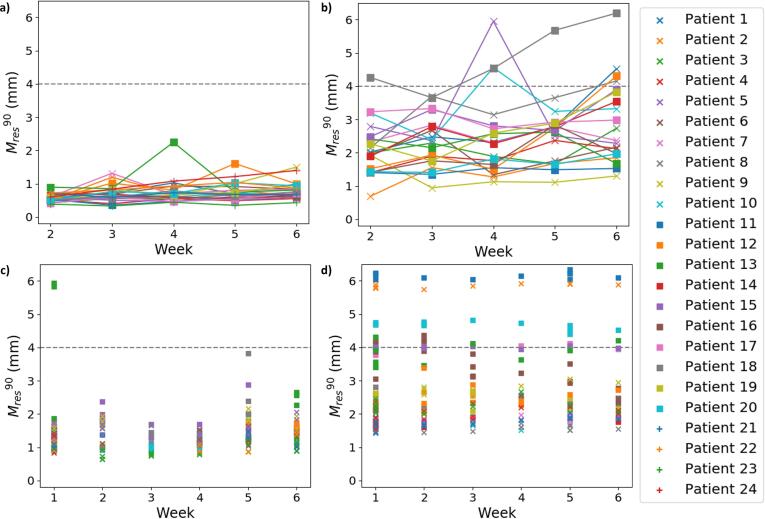
The 90th percentile of $M_{\mathrm{res}}$ calculated on one DVF from each of weeks 2–6 of treatment for each patient for the patient-specific models created using a) dataset 1, b) dataset 2, and for the population-based model calculated on all DVFs not used for model creation from each of weeks 1–6 using the c) training patients (dataset 3a), d) validation patients (dataset 3b).

**Fig. 4:**
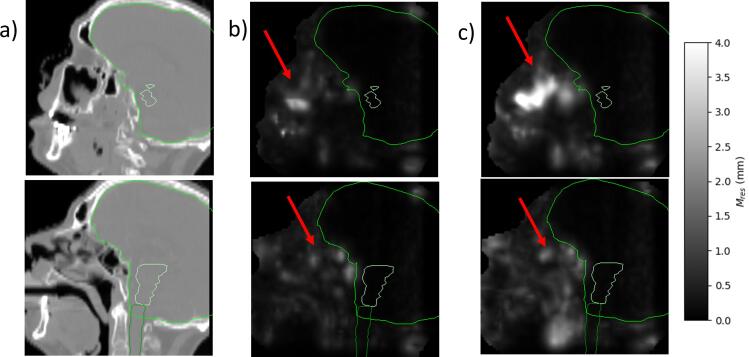
Identifying areas with a high $M_{\mathrm{res}}$ for the patient-specific model (patient 1, dataset 1). The panels show a) the pCT for a patient in two different slices (top showing nasal cavity region and bottom showing the oropharyngeal region), b) the mean of $M_{\mathrm{res}}$ from LOOCV and c) the mean of $M_{\mathrm{res}}$ from the following weeks. Contours of the brain, brainstem and spinal cord are shown in green. (For interpretation of the references to colour in this figure legend, the reader is referred to the web version of this article.)

The ability of the patient-specific models created with the DVFs from week 1 to adequately describe deformations present later during treatment is shown in Fig. 3. Most of the patients passed the 4 mm threshold in subsequent weeks. As expected, a tendency for ${M_{\mathrm{res}}}^{90}$ to increase over time during the treatment course was observed. Using DVFs from the first two weeks resulted in slightly decreased numbers, but with the same trend over time (see data in Supplement 5). It should be noted that two of the patients who failed the generalisability evaluation also failed the LOOCV (patients 10/18). A heat map of the mean (across all DVFs used for investigating the model generalisability) $M_{\mathrm{res}}$ for patient 1 of dataset 1 is shown in Fig. 4 (remaining patients shown in Supplement 6). The largest $M_{\mathrm{res}}$ values were found in the oropharynx region for all patients, and additionally in the nasal cavities for dataset 1 patients.

**Fig. 5:**
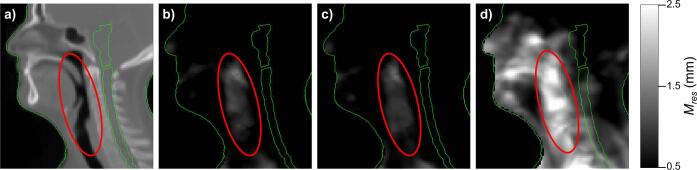
Identifying trends in areas with a high $M_{\mathrm{res}}$ for the population-based model for both training and validation patients. The panels show a) the average pCT, b) the mean of $M_{\mathrm{res}}$ from the LOOCV (120 DVFs), c) the mean of $M_{\mathrm{res}}$ from all the DVFs not used to create the PCA model of the training patients (217 DVFs) and d) the mean of $M_{\mathrm{res}}$ from all the DVFs of the validation patients (254 DVFs). The region with a consistently high $M_{\mathrm{res}}$ is outlined in red. The external contour and the brainstem and spinal cord are shown in green for context. (For interpretation of the references to colour in this figure legend, the reader is referred to the web version of this article.)

For the population-based model, there was little difference in performance between different treatment weeks, but the model performed worse for validation patients than training patients. Three of the validation patients were above the 4 mm threshold for all DVFs evaluated (Fig. 3). For the training patients, 2 DVFs from the first week of treatment for a single patient failed to reach the threshold (who also had the second highest value for the LOOCV in week 1). $M_{\mathrm{res}}$ was largest around the oropharynx, consistent with the patient-specific results (Fig. 5). The mean (across all available DVFs) $M_{\mathrm{res}}$ values were higher for the validation patients in this and surrounding regions. Around the brainstem and within the skull, $M_{\mathrm{res}}$ was consistently low for both the training and validation patients.

A scatter plot linking the mean ${M_{\mathrm{res}}}^{90}$ for the LOOCV with the mean ${M_{\mathrm{res}}}^{90}$ for the generalisability is presented in Supplement 7.

## 4 Discussion

We have presented a toolbox to evaluate how well anatomical changes in unseen data can be described by PCA models and tested it for H&N cancer patients in both patient-specific and population-based contexts. Our evaluation strategy can be used to easily determine patients where PCA modelling could be used (e.g., by applying LOOCV and setting up a threshold of acceptable error) and highlight anatomical regions where the PCA model struggles.

To the best of our knowledge, no other study has proposed such a method. Similar evaluation strategies have focussed on either determining the required number of components to use in a PCA model [6], [9], [10], [12] or the optimal number of input scans needed to create the model [9]. Generally, these strategies tend to evaluate the PCA model against input DVFs, whereas our method compares the model to unseen DVFs. However, Badawi et al. [9] evaluated the residual error on unseen scans for PCA models created using a subset of the input scans, but this was mainly used to assess how many weeks’ worth of input scans was needed for the residual errors to stabilise. Budiarto et al. [12] also evaluated the residual error on three patients not previously seen by their population-based model, but did not evaluate on any unseen DVFs from their training patients. For our population-based model, the validation patients performed worse in the evaluation than the training patients (see Supplement 8).

Some studies using PCA looked at deformations to the surface contour of specific organs as inputs to the model [9], [10], [12], while others considered each image voxel [7], [14], [22]. Here we have used the voxel-based approach as this allows us to model changes on the entire scan and include multiple organs and their interactions, as well as other surrounding tissue.

Our scheme focuses on computing the magnitude difference between the reference and closest DVF and does not differentiate between differences in magnitude and direction for each vector. Of course, one could also evaluate each of the ×, y and z directions separately.

While the method only evaluates individual instantiations of a DVF, by combing multiple results, the toolbox can be used to assess the quality of the model. E.g., by computing $M_{\mathrm{res}}$ for multiple known DVFs, this can give an idea of what modes of deformations are/are not captured by the model. In addition, our method also calculates the Z-score for each component, providing an idea of the magnitude of the deformations captured. Providing Z-scores across all sampled DVFs follow a normal distribution, the model accurately captures the size of the deformations sampled and will correctly describe expected changes.

This work relies on PCA, so all limitations related to this method will apply. A vital part of PCA modelling and the evaluation toolbox are the deformable registrations. Although registrations were visually inspected to ensure quality, they will still introduce uncertainties into both the PCA model and residual DVFs. For this study, a quantitative assessment of registration accuracy was not done, but we would recommend this for specific applications depending on the required accuracy. To give an idea of the possible size of these uncertainties, Veiga et al. [23] found the mean distance transform between manual and deformed surfaces for H&N registrations using NiftyReg to be 0.3 ± 0.4 mm. We assumed that any uncertainties associated with imperfect registrations would be smaller than the anatomical changes we aim to capture in the model – e.g., Barker et al. [24] found the median medial shift of the centre of mass of the parotid throughout treatment to be 3.1 mm.

Previous studies have investigated the generation of PCA models in H&N cancer patients and their evaluation. For example, Tsiamas et al. [15], assessed the number of components needed to model the spatial displacements for specific organs, using data from 18 patients to create both individual and population-based models. They focused on comparing the relative variance of the different PCA components between models. They found three to four principal components were sufficient to achieve spatial displacement prediction at the 95% confidence level for normal organs. In another study, Chetvertkov et al. [16] generated PCA models considering variations occurring in the whole scan in 10 patients. They focused on whether the weighting factors from the inner product could be used to predict systematic changes throughout the treatment course, and dismissed the errors not expressed by linear combinations of the eigenvectors. Our results show that these errors can be considerable and should not be dismissed.

The patient-specific models were created from scans from the first week of treatment and could for instance be used either for informing a patient-specific threshold for adaptation, or for including variations seen in the PCA model in a patient-specific robust optimization. In the first case, a set of possible patient geometries could be simulated from the model, dosimetric evaluation could be performed, and an assessment of whether adaptation is required be made. In the second case, a robust plan could be made to include the variations seen in the PCA model, such that within this envelope of variations no adaptation would be required for the rest of the treatment. Our population-based model was created using treatment scans across 6 weeks from 20 patients, providing the opportunity to use it to simulate possible anatomical changes on new patients, without having to wait for multiple scans. This means population-based models could be used at the planning stage to evaluate the robustness of treatment plans against expected anatomical changes or to directly account for them by using robust or probabilistic planning. In this setting, our evaluation scheme could provide information on residual uncertainties that could be used to generate additional scenarios. Depending on the specific application, PCA models may be tuned to focus either on systematic variations (seen in the lower order components) and/or random variations (in higher order components).

We observed that ${M_{\mathrm{res}}}^{90}$ varied between datasets. One possibility is that the oropharynx tumours in datasets 2 and 3 may affect the model performance as it changes the anatomy and the tumour may shrink during treatment. Another possibility is the difference in bounding boxes between patient datasets. The bounding box for dataset 1 was based on a region surrounding the skull, meaning there was likely to be better anatomical consistency than for datasets 2 and 3, where the bounding boxes generally extended further down the neck which is prone to larger deformations [25]. This shows that the residual error depends on the region of interest being considered, which should be carefully selected to cover clinically relevant organs. One way to eliminate the bias of the bounding box could be to report ${M_{\mathrm{res}}}^{90}$ for the target volume and the relevant organs individually.

By using our evaluation toolbox, regions where a PCA model struggles to represent deformations can easily be identified. For example, the oropharynx presented a challenge for both the patient-specific models (using 5/10 input DVFs) and the population-based model (using 120 input DVFs). This suggests that it is not a limit in the number of scans that is causing this challenge, but rather a limitation of the PCA method itself. PCA assumes variations are normally distributed around a non-zero mean. This assumption is commonly seen in the literature for models for day-to-day changes in organ shape [13], [14], but it has not been validated in H&N. Additionally, for the sinonasal cancer patients, the nasal cavity was highlighted as a challenging region. This is due to the cavity being filled/emptied, which violates the assumption of the variation of the position of each voxel being normally distributed.

In conclusion, we have proposed and tested a toolbox to evaluate how well PCA models can predict anatomical deformations. We showed how regions were identified where models created from the first week of radiotherapy in H&N cancer patients struggle to represent anatomical changes occurring later during treatment. All tested models had difficulties capturing deformations in the oropharyngeal region, and the nasal cavity for models created on sinonasal cancer patients. Our methods could potentially be used in a variety of scenarios to evaluate and validate PCA models and facilitate incorporation of deformation modelling in various clinical applications.

## Declaration of Competing Interest

The authors declare that they have no known competing financial interests or personal relationships that could have appeared to influence the work reported in this paper.
