# Supplementary material for: Evaluating principal component analysis models for representing anatomical changes in head and neck radiotherapy
Source: Phys Imaging Radiat Oncol. 2022 Apr 13;22:13–9. doi: 10.1016/j.phro.2022.04.002 (PMC9038571; doi:10.1016/j.phro.2022.04.002)
Supplement: Supplementary data 1 [file mmc1.pdf]

Supplement 1

To get an indication of how likely it is for $\overline{\boldsymbol{v}}_{closest}$ to occur from the PCA model, we calculated the Z-score (the weight divided by $\sqrt{var_i}$) for the weight of each component, and report the maximum Z-score, Z_{max}, for each DVF. Following the empirical rule, if the Z-score is less than 3, there is more than 99% chance that it could have been selected from the model. For models with a large number of components the percentage of Z-scores under 3 can also be reported. If this number is significantly less than 99.7%, it is unlikely that $\overline{\boldsymbol{v}}_{closest}$ could be randomly produced from the model.

Robustness (LOOCV):

Z_{max} ranged from between 0.1-1.6 for dataset 1 and between 0.1-1.8 for dataset 2. For the population-based study (dataset 3a), Z_{max} ranged from 1.8-5.7. 99.2% of all the Z-scores were below 3.

Generalisability:

For the DVFs not used to train the PCA models, Z_{max} ranged between 0.2-1.8 for dataset 1 and 0.2-2 for dataset 2. For the population-based datasets, Z_{max} ranged from 1.7-13.0 for the training patients with 99.0% of the Z-scores below 3 and for the validation patients, Z_{max} ranged from 2.0-17.5 with 91.6% of the Z-scores below 3. The DVFs with the highest Z_{max} also tended to be those with high ${M_{res}}^{90}$ in the population-based model.

Supplement 2

We evaluated the proposed PCA method in simulated vector fields, $\overline{\boldsymbol{v}}_{sim}$, randomly created using equation (1).

$$\overline{\boldsymbol{v}}_{sim} = \overline{\boldsymbol{v}}_{mean} + \textstyle\sum_i u_i \overline{\boldsymbol{e}}_i \quad (1)$$

We assessed the robustness of the evaluation strategy defined in section 2.1 of the paper, by quantifying the impact of adding differing levels of Gaussian noise on $\overline{\boldsymbol{v}}_{sim}$. We randomly produced 50 $\overline{\boldsymbol{v}}_{sim}s$ and added spatially independent Gaussian noise (standard deviation, SD, of 0, 1, and 2 mm) to each vector. For each simulated DVF (including noise), we calculated ${M_{res}}^{90}$ using the first c components from the PCA model, where $1 \le c \le n\text{-}1$, the total number of components. These ${M_{res}}^{90}$ values were plotted against c, along with the mean for all 50 DVFs and are shown in Supplementary Figure S1.

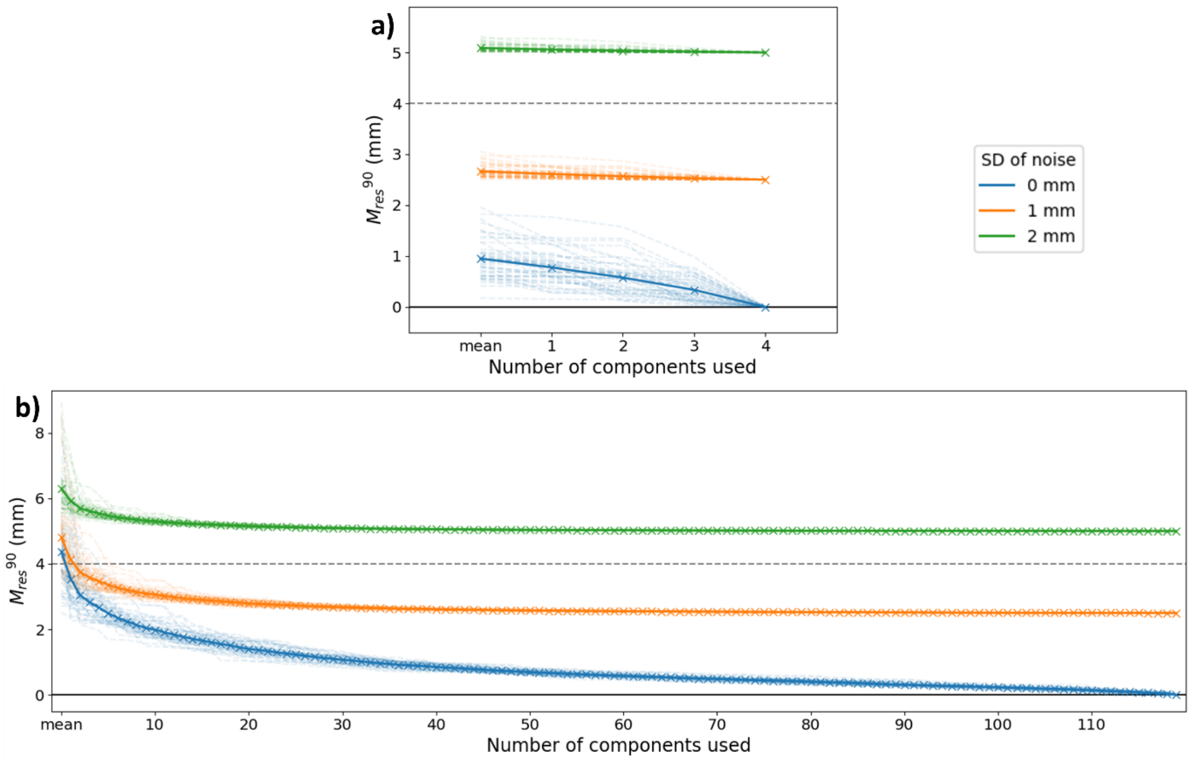

Supplementary Figure S1. Comparing $M_{res}{}^{90}$ for DVFs created from the PCA model with differing levels of noise, analysed using a different number of components from the model. The dashed lines show the results from each of 50 randomly generated DVFs and the solid line shows the mean of those 50. Plot a) shows the patient-specific model (patient 1, dataset 2) and plot b) shows the population-based model.

The tests with the simulated data (Supplementary Figure S1) show that for all noise-free simulated DVFs, $M_{res}{}^{90}$ (and actually all M_{res}) reached zero after all components were accounted for, validating our evaluation approach. We also established that adding Gaussian noise with SD of 2 mm pushed $M_{res}{}^{90}$ above our chosen threshold of 4 mm. Regardless of the level of Gaussian noise, the Z_{max} values were the same and ranged between 0.3-2.6 for all patient-specific models and 1.9-3.9 for the population-based model. 99.7% of all the Z-scores were below 3 for the population-based model.

Supplement 3

Patient datasets are detailed below:

Dataset 1: Data from twenty-four sinonasal cancer patients treated either with primary or postoperative photon therapy (IMRT) with curative intent from 2009 to 2017 were included

in this dataset. The data was collected from the DAHANCA database (approval by the Danish Data Protection Agency (1-16-02-676-18)). These patients were treated to a prescribed dose to the primary CTV of either 66-68 Gy for primary radiotherapy or 60-66 Gy for post-operative radiotherapy. Patients had between 30-34 CBCTs.

Dataset 2: Data from twenty oropharyngeal cancer patients treated in the period of 2008-2018 with radical radiotherapy in a single institution were arbitrarily selected. The data was fully anonymised and collected from a clinical database after internal study approval at the clinical department, in accordance with Danish guidelines. Ten patients were treated with IMRT and ten with volumetric-modulated arc therapy (VMAT), seventeen to a prescribed dose of 66-68 Gy (33-34 fractions, 5-6 per week) and three to a prescribed dose of 76 Gy (56 fractions, 10 per week). Patients had between 33-56 CBCTs.

Dataset 3: Data from forty oropharyngeal patients treated between 2016 and 2018 with radical radiotherapy in a single institution were selected. These data were collected from the ukCAT distributed learning database (ethics approval from the UK North West - Haydock Research Ethics Committee, (17/NW/0060), local consent ref. 2018-018). All patients were treated in 30 fractions using VMAT to a prescribed dose of 60-66 Gy to the primary CTV. Twenty patients were selected as the training patients, denoted dataset 3a. The remaining twenty patients were used for validation and denoted dataset 3b. Patients had between 8-31 CBCTs.

Supplement 4

Here we report the cumulative variance for each component in the PCA models for the first patient-specific model in each of datasets 1 and 2 and for the population-based model.

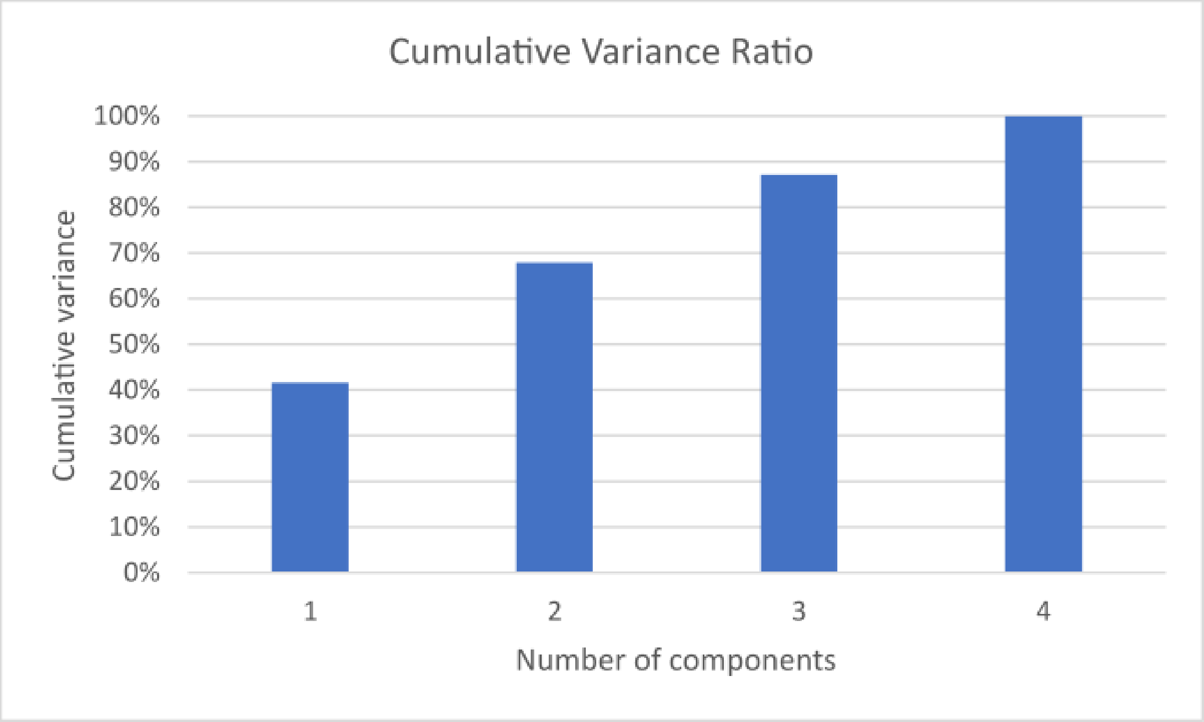

Supplementary Figure S2. Cumulative variance ratio of each component for patient 1 of dataset 1.

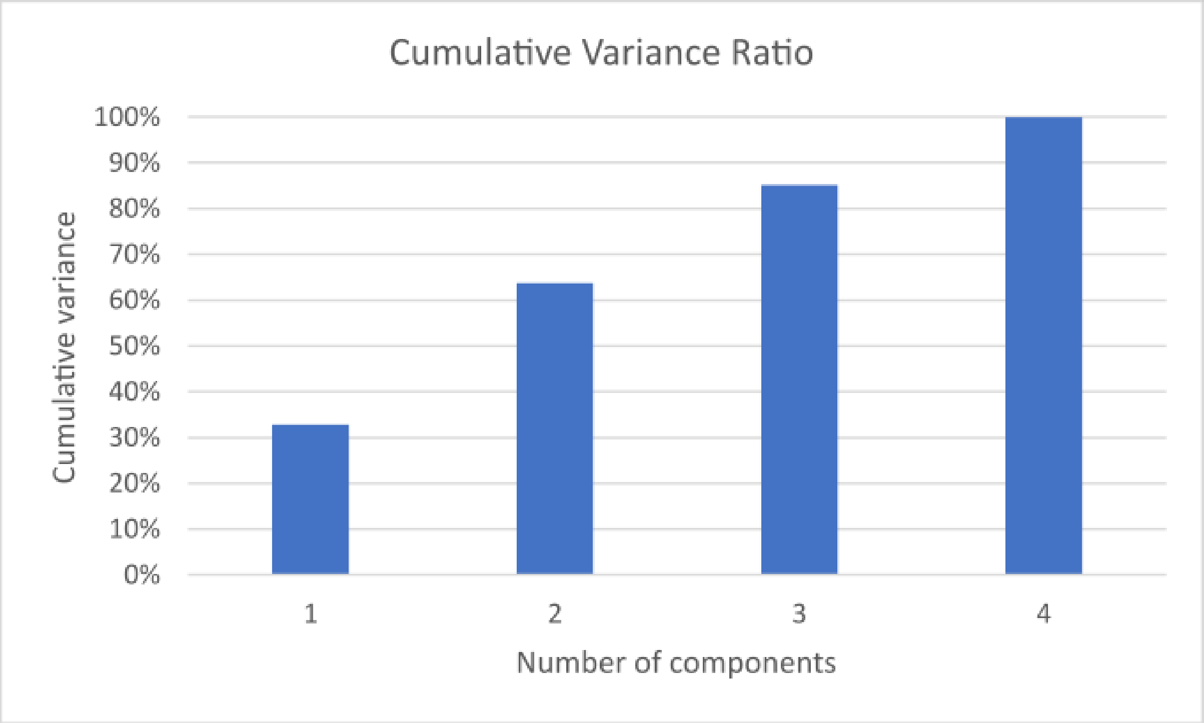

Supplementary Figure S3. Cumulative variance ratio of each component for patient 1 of dataset 2.

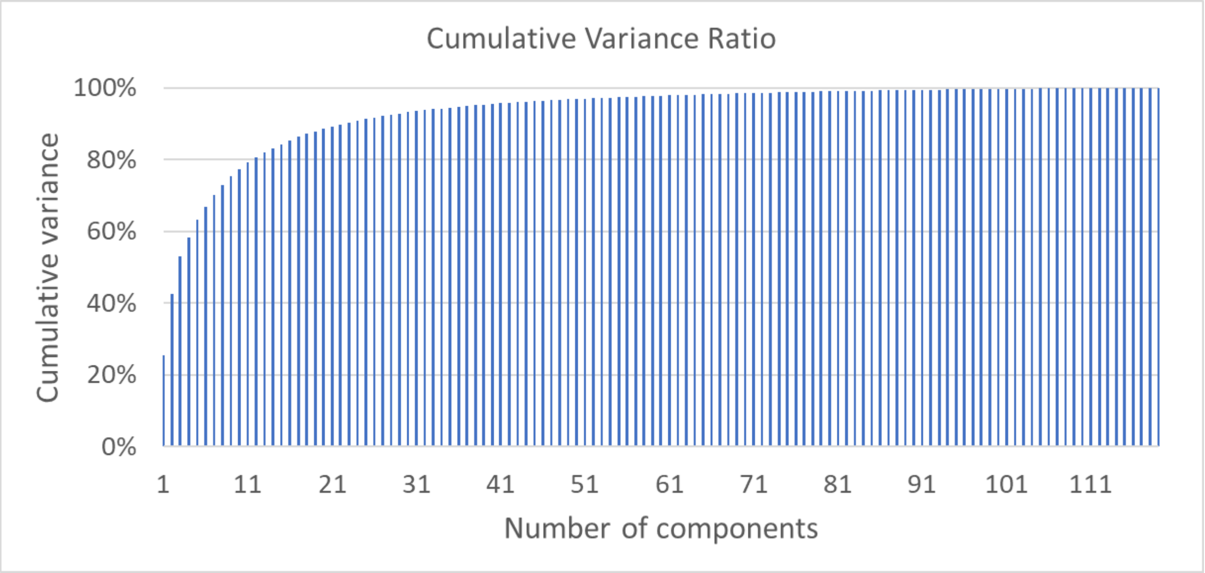

Supplementary Figure S4. Cumulative variance ratio of each component for dataset 3a.

Supplement 5

As shown in the figures below, increasing the number of input scans will improve the model and decrease the M_{res} values , but the same region is still highlighted as posing a challenge for the models. This suggests that it is perhaps a fundamental limitation of the PCA method that is causing the difficulties in modelling the changes within the oropharynx.

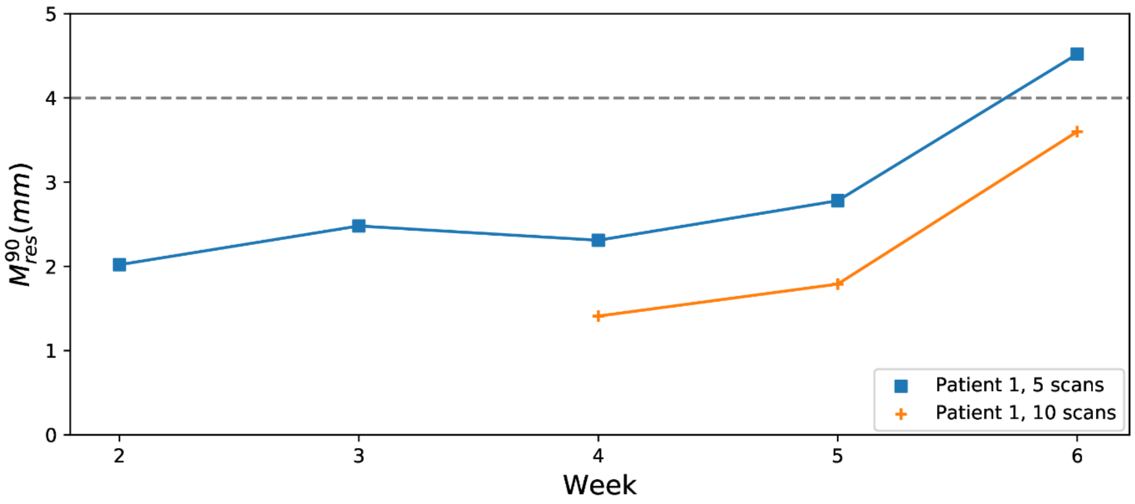

Supplementary Figure S5. $M_{res}{}^{90}$ calculated on the later weeks for patient 1 (dataset 2) with the model created: using the DVFs from first week (blue) and using the DVFs from first two weeks (orange).

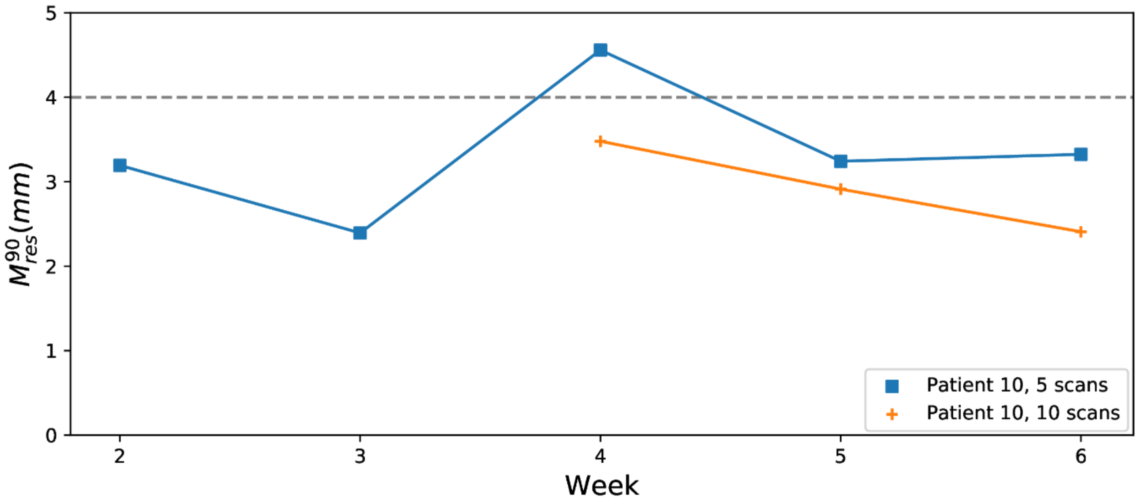

Supplementary Figure S6. $M_{res}{}^{90}$ calculated on the later weeks for patient 10 (dataset 2) with the model created: using the DVFs from first week (blue) and using the DVFs from first two weeks (orange).

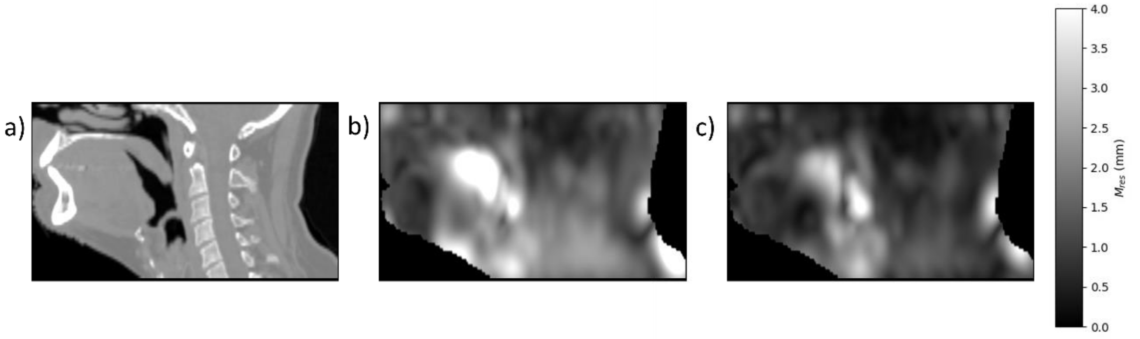

Supplementary Figure S7. Identifying areas with high M_{res} for patient 1 (dataset 2). The panels show a) the pCT b) the mean of M_{res} from the later weeks with model created with the DVFs from first week c) the mean of M_{res} from the later weeks with model created with the DVFs from first two weeks.

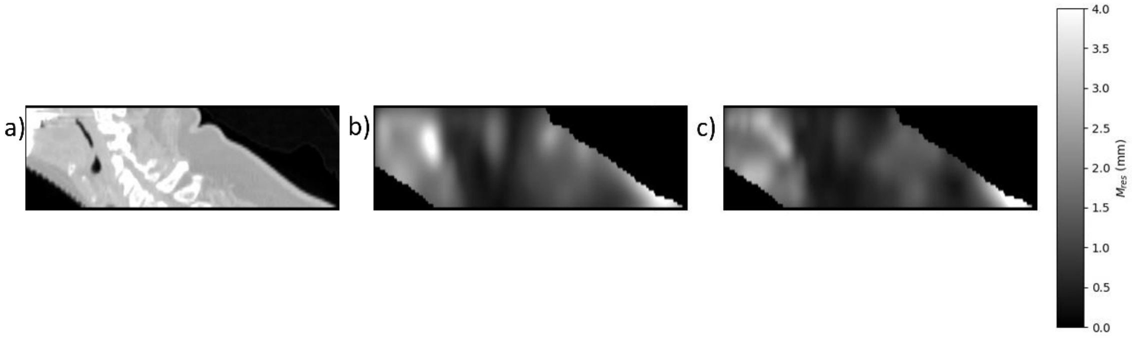

Supplementary Figure S8. Identifying areas with high M_{res} for patient 10 (dataset 2). The panels show a) the pCT b) the mean of M_{res} from the later weeks with model created with the DVFs from first week c) the mean of M_{res} from the later weeks with model created with the DVFs from first two weeks.

Supplement 6

The following figures show the heat maps of M_{res} for all patient-specific cases. The panels show a) the pCT, b) the mean M_{res} for the LOOCV and c) the mean M_{res} for the following weeks.

Dataset 1:

Patient 1

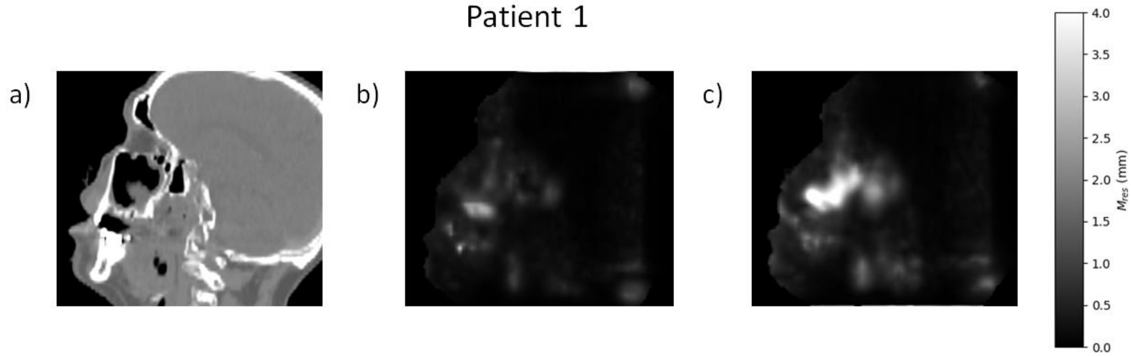

Patient 2

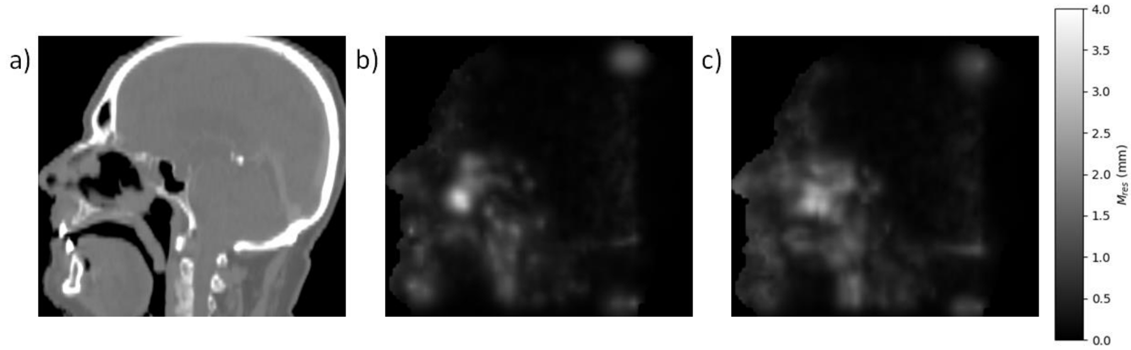

Patient 3

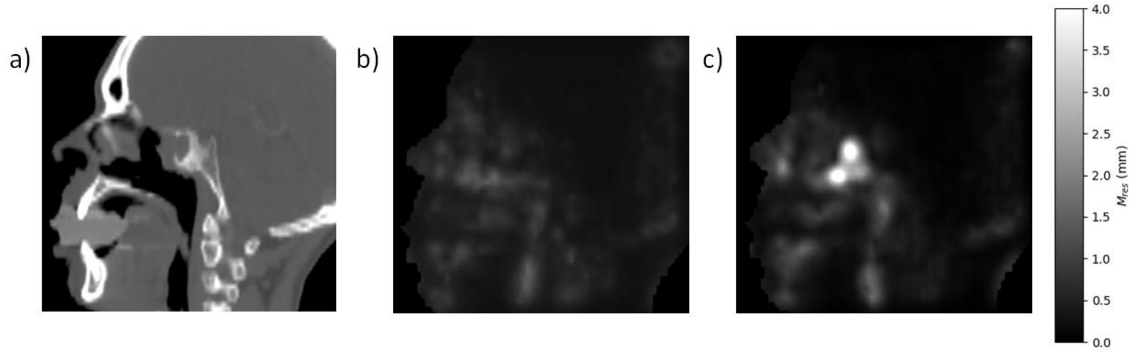

Patient 4

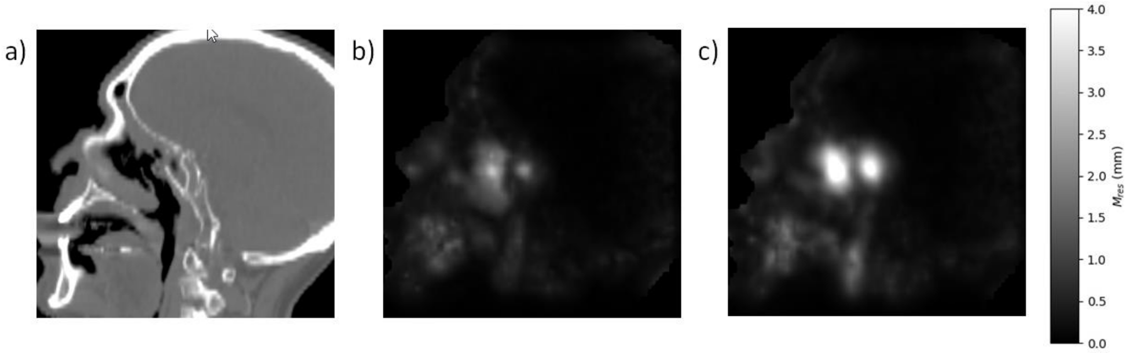

Patient 5

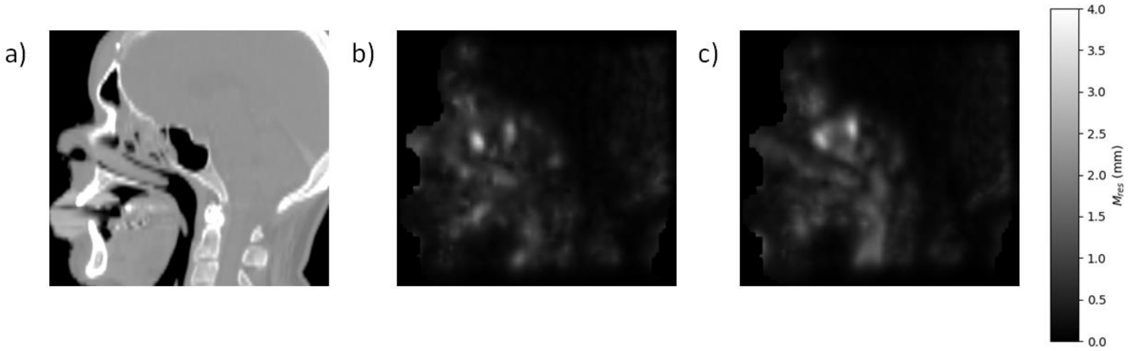

Patient 6

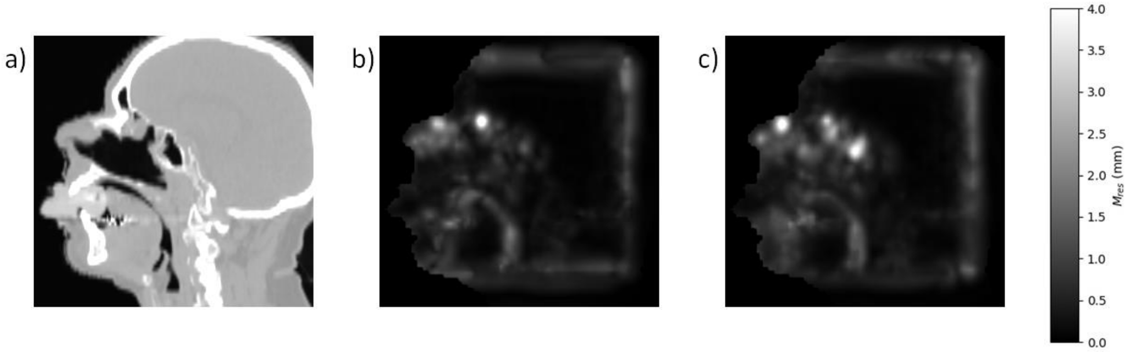

Patient 7

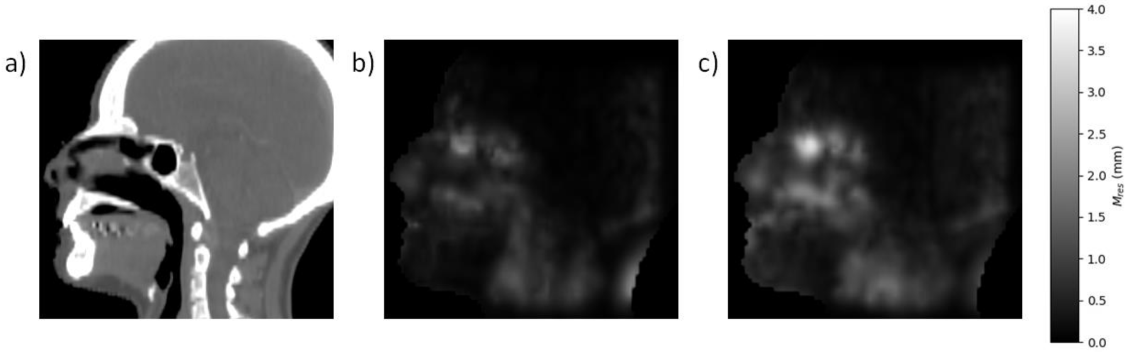

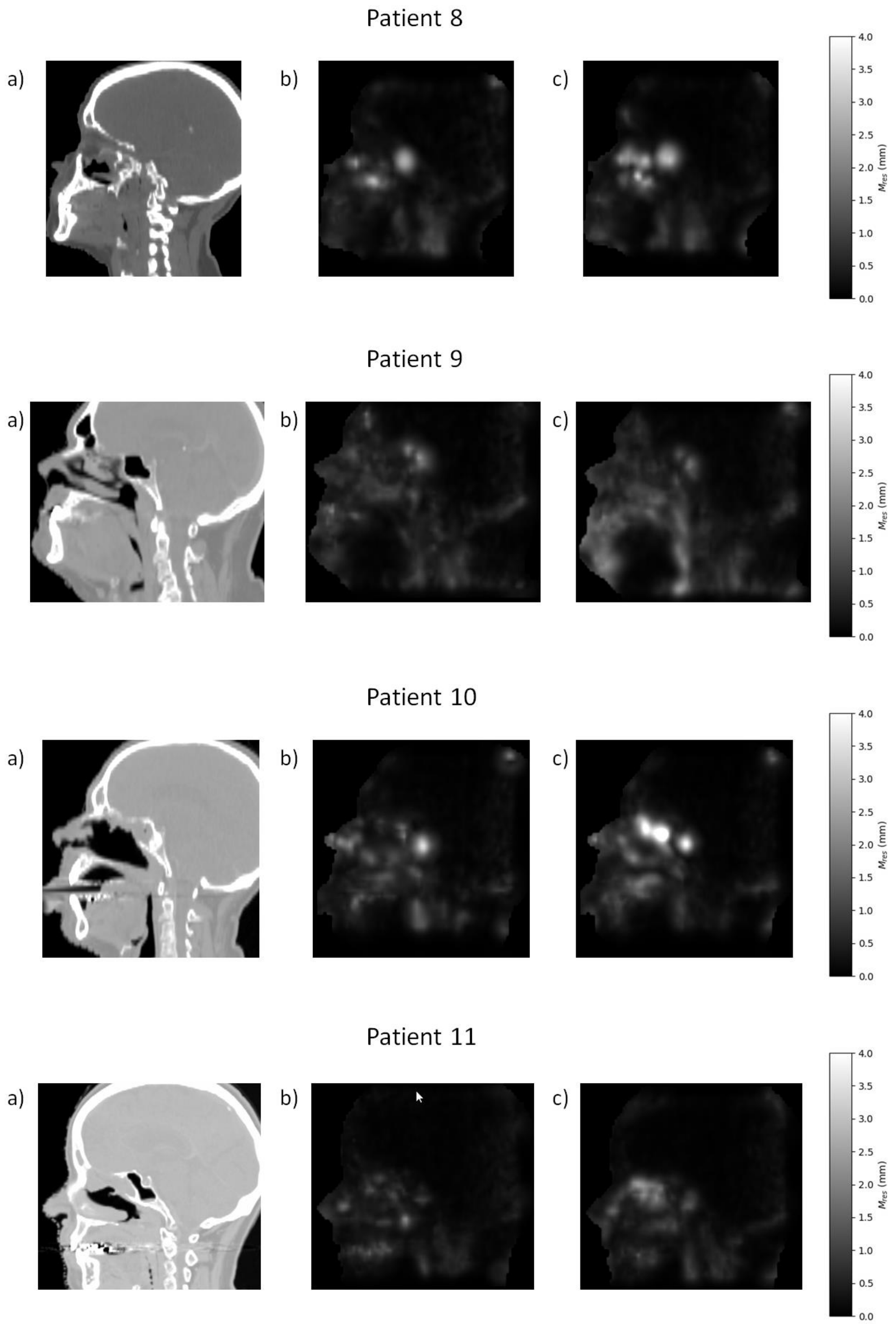
Patient 8
a)
b)
c)
Patient 9
a)
b)
c)
Patient 10
a)
b)
c)
Patient 11
a)
b)
c)
0.0
0.5
1.0
1.5
2.0
2.5
3.0
3.5
4.0
M_{res} (mm)

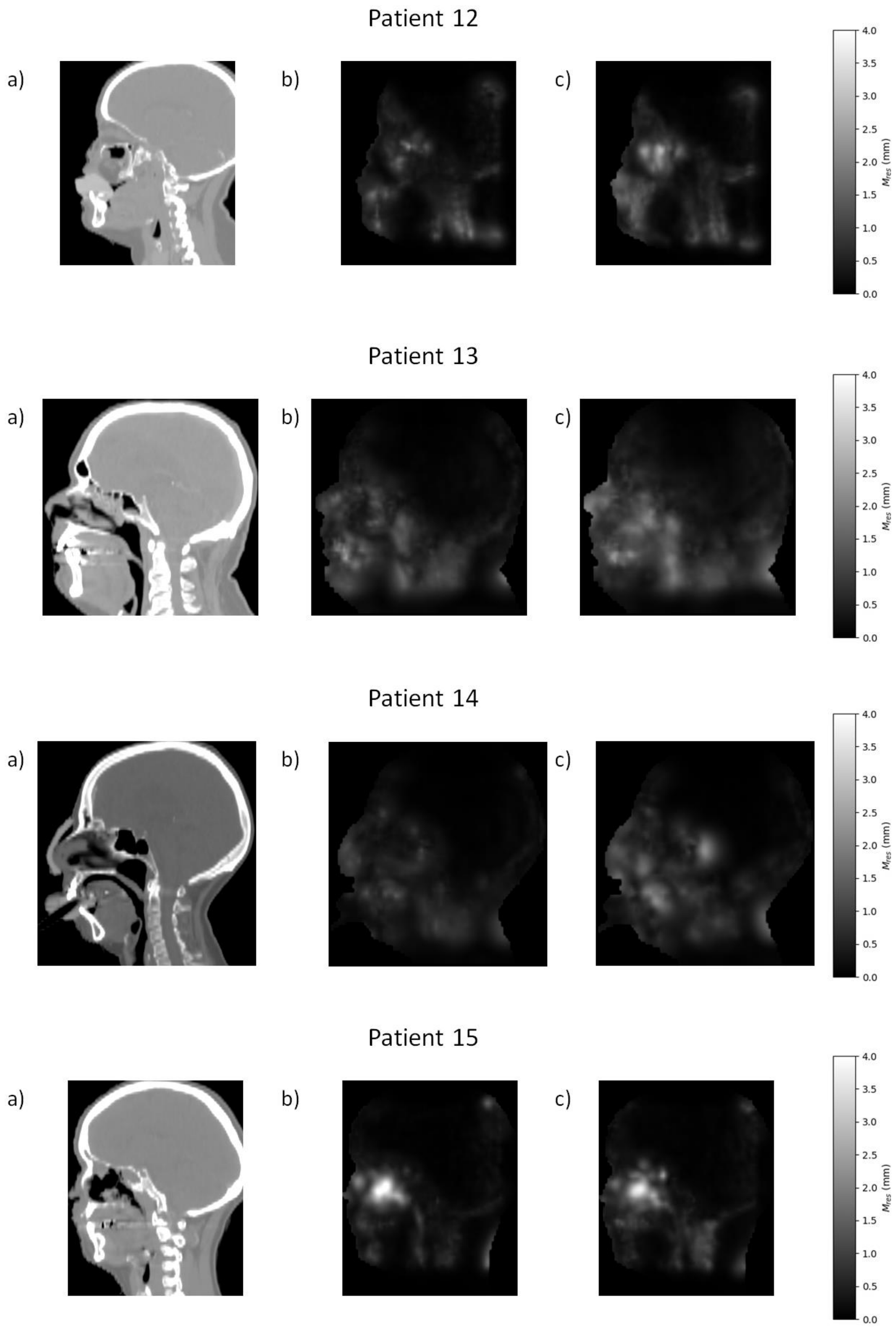

Patient 12
a)
b)
c)
4.0
3.5
3.0
2.5
2.0
1.5
1.0
0.5
0.0
M_{res} (mm)
Patient 13
a)
b)
c)
4.0
3.5
3.0
2.5
2.0
1.5
1.0
0.5
0.0
M_{res} (mm)
Patient 14
a)
b)
c)
4.0
3.5
3.0
2.5
2.0
1.5
1.0
0.5
0.0
M_{res} (mm)
Patient 15
a)
b)
c)
4.0
3.5
3.0
2.5
2.0
1.5
1.0
0.5
0.0
M_{res} (mm)

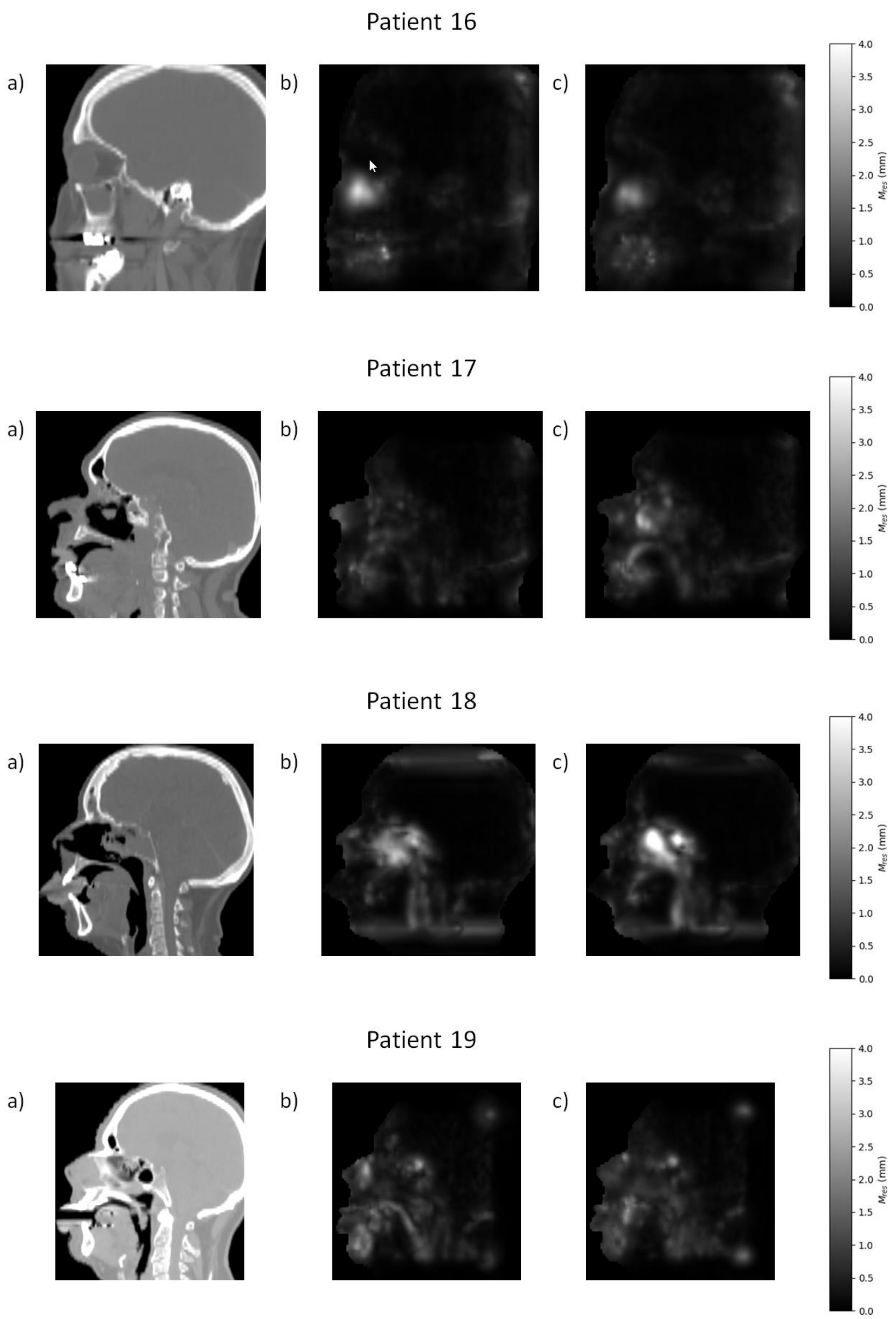
Patient 16
a)
b)
c)
Patient 17
a)
b)
c)
Patient 18
a)
b)
c)
Patient 19
a)
b)
c)
0.0
0.5
1.0
1.5
2.0
2.5
3.0
3.5
4.0
M_{res} (mm)

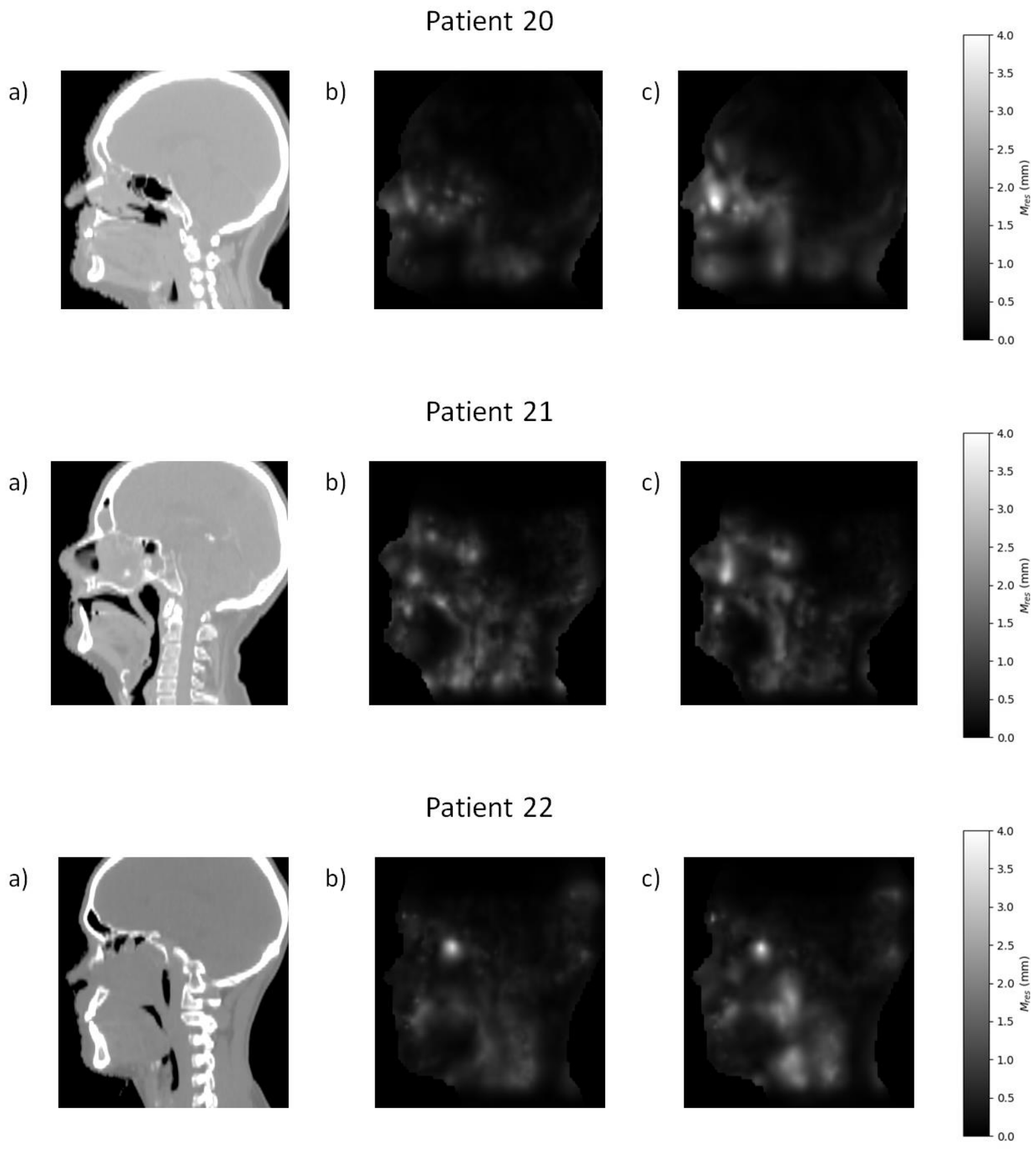
Patient 20
a)
b)
c)
Patient 21
a)
b)
c)
Patient 22
a)
b)
c)
4.0
3.5
3.0
2.5
2.0
1.5
1.0
0.5
0.0
M_{res} (mm)

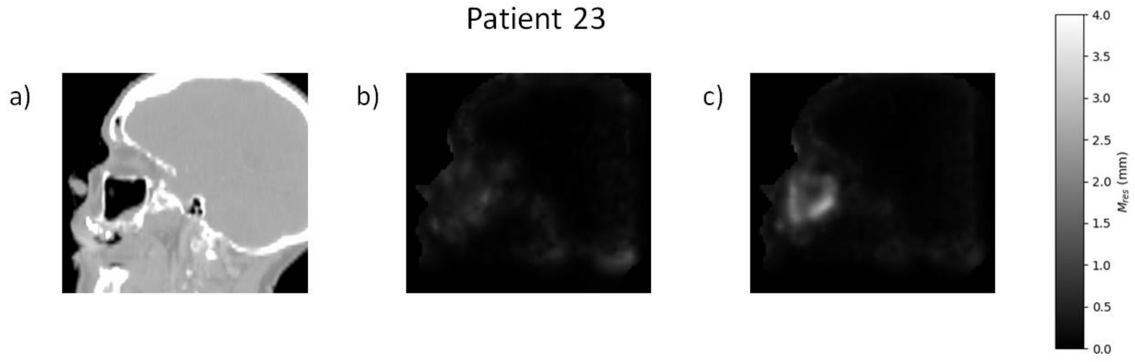
Patient 23
a)
b)
c)
M_{res} (mm)
0.0
0.5
1.0
1.5
2.0
2.5
3.0
3.5
4.0

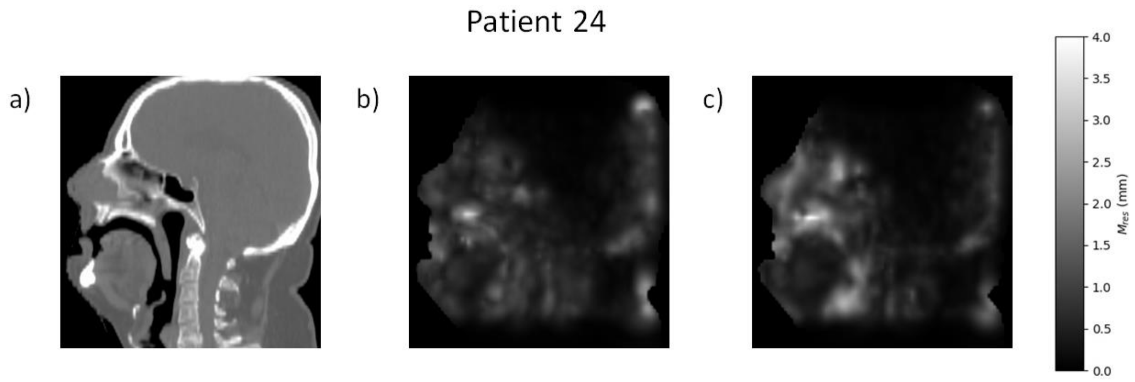
Patient 24
a)
b)
c)
M_{res} (mm)
0.0
0.5
1.0
1.5
2.0
2.5
3.0
3.5
4.0

Dataset 2:

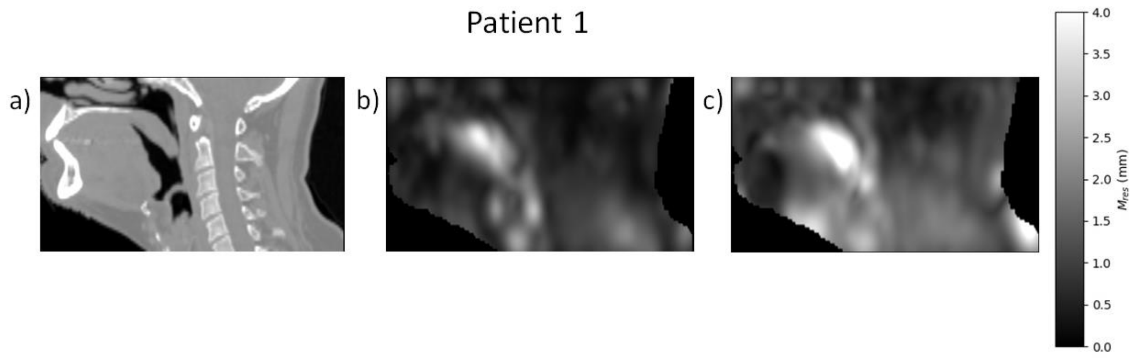
Patient 1
a)
b)
c)
M_{res} (mm)
0.0
0.5
1.0
1.5
2.0
2.5
3.0
3.5
4.0

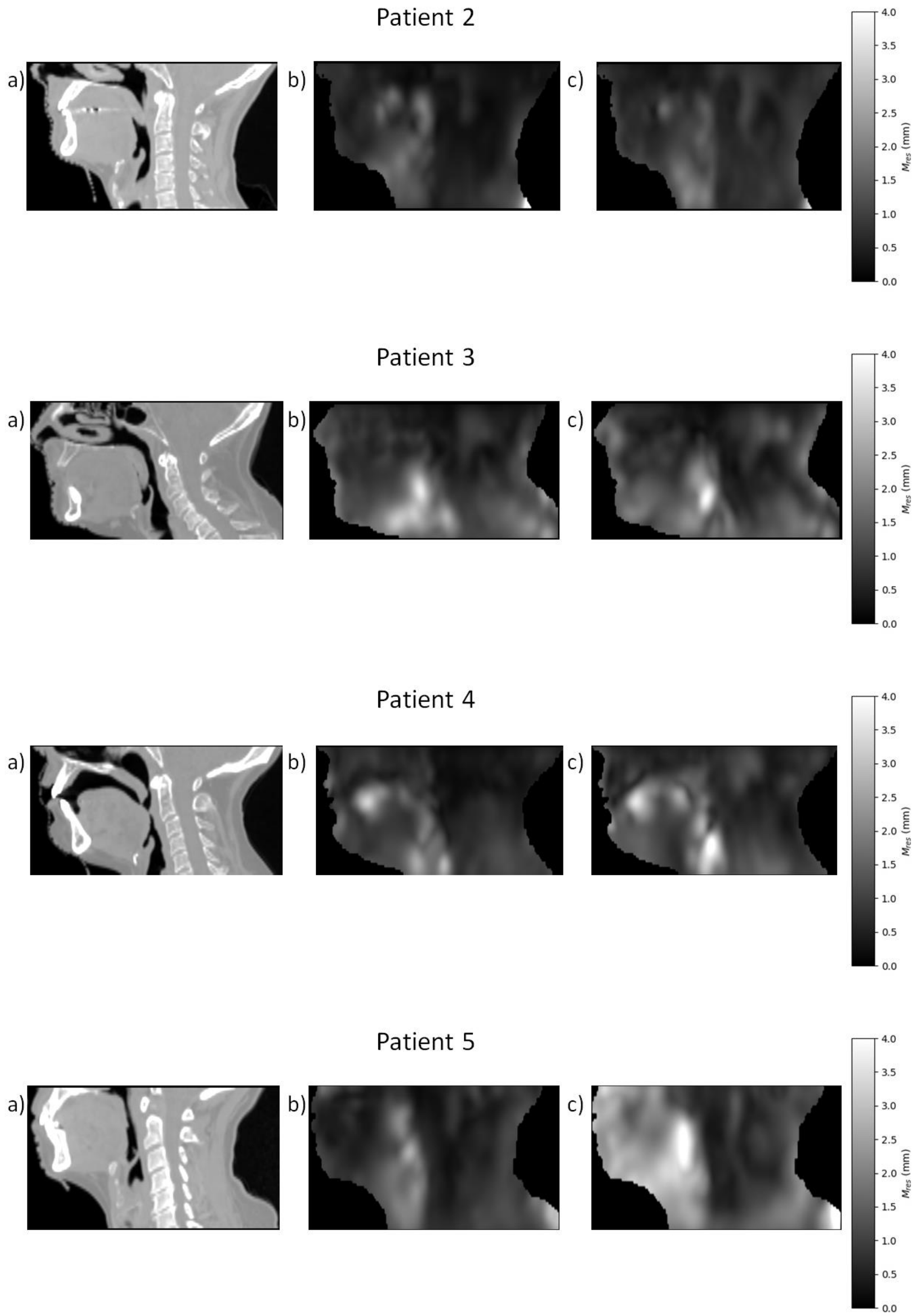
Patient 2
a)
b)
c)
Patient 3
a)
b)
c)
Patient 4
a)
b)
c)
Patient 5
a)
b)
c)
4.0
3.5
3.0
2.5
2.0
1.5
1.0
0.5
0.0
M_{res} (mm)

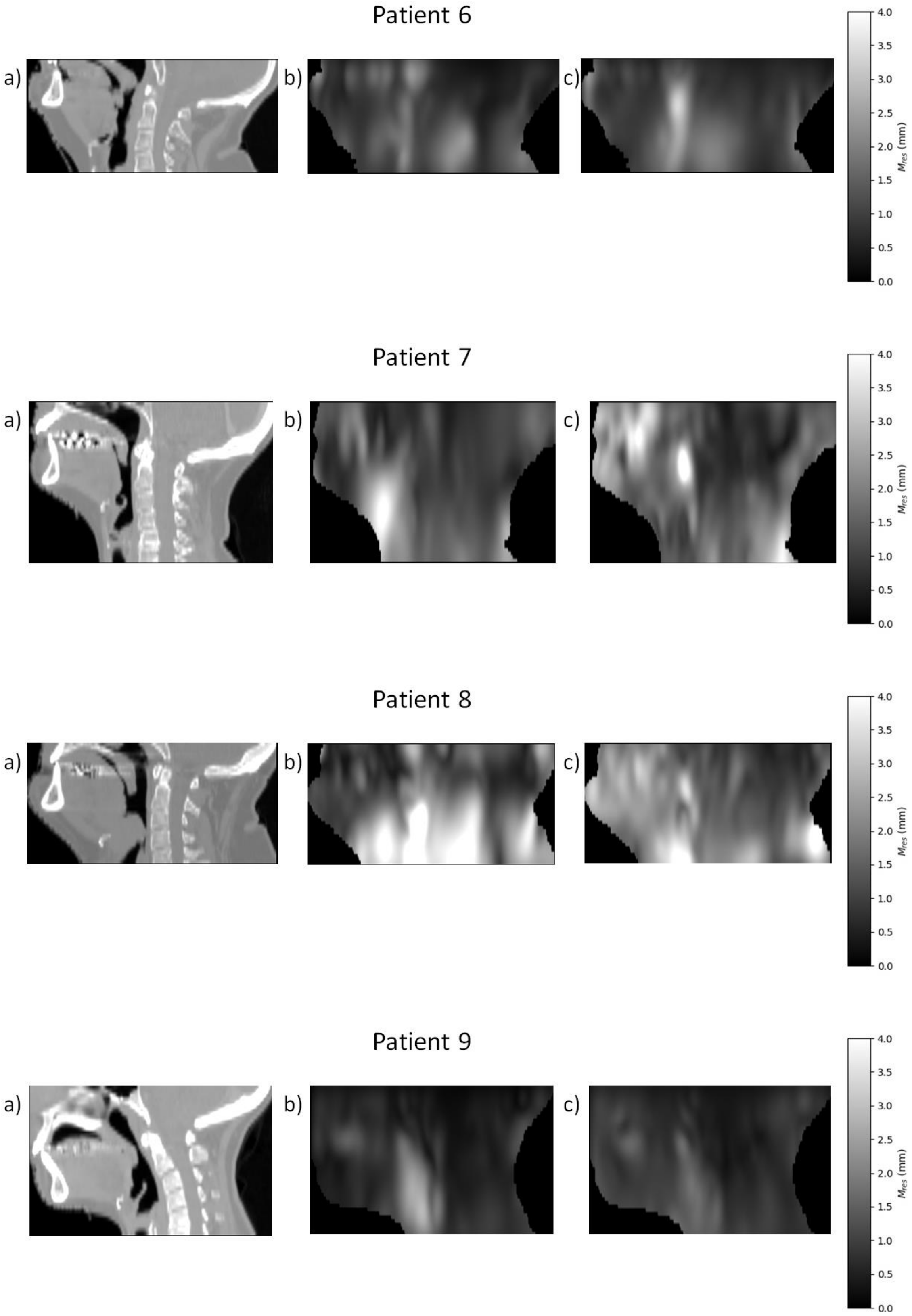

Patient 6
a)
b)
c)
Patient 7
a)
b)
c)
Patient 8
a)
b)
c)
Patient 9
a)
b)
c)
4.0
3.5
3.0
2.5
2.0
1.5
1.0
0.5
0.0
M_{res} (mm)

Patient 10

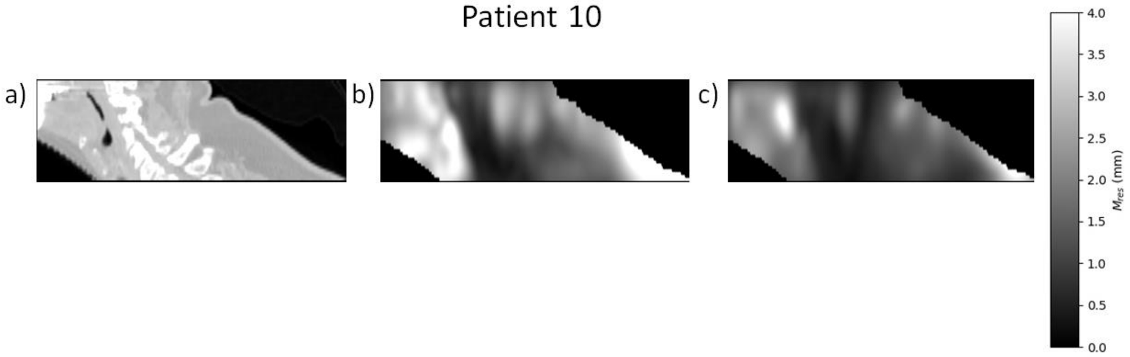

Patient 11

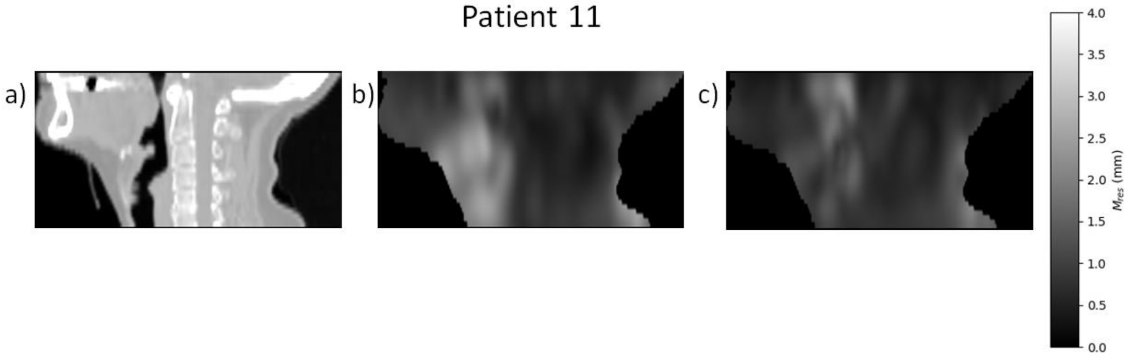

Patient 12

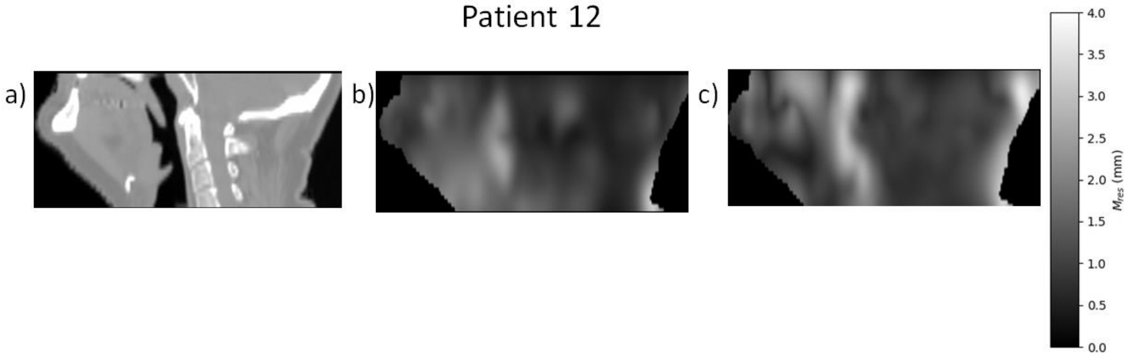

Patient 13

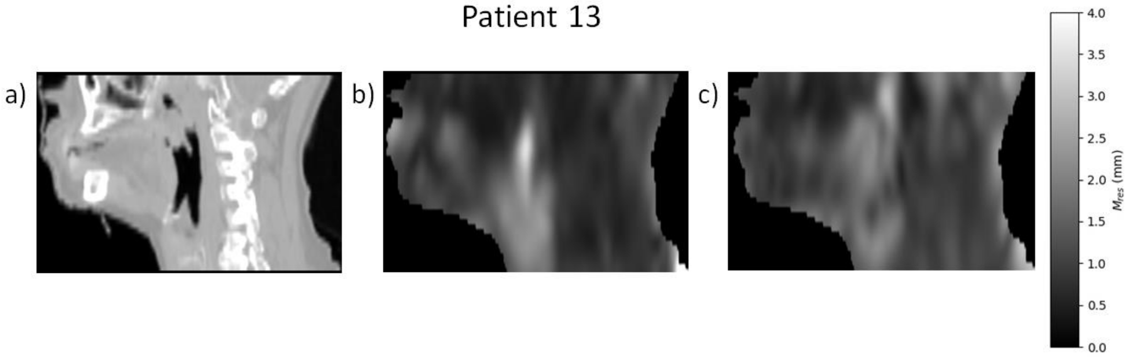

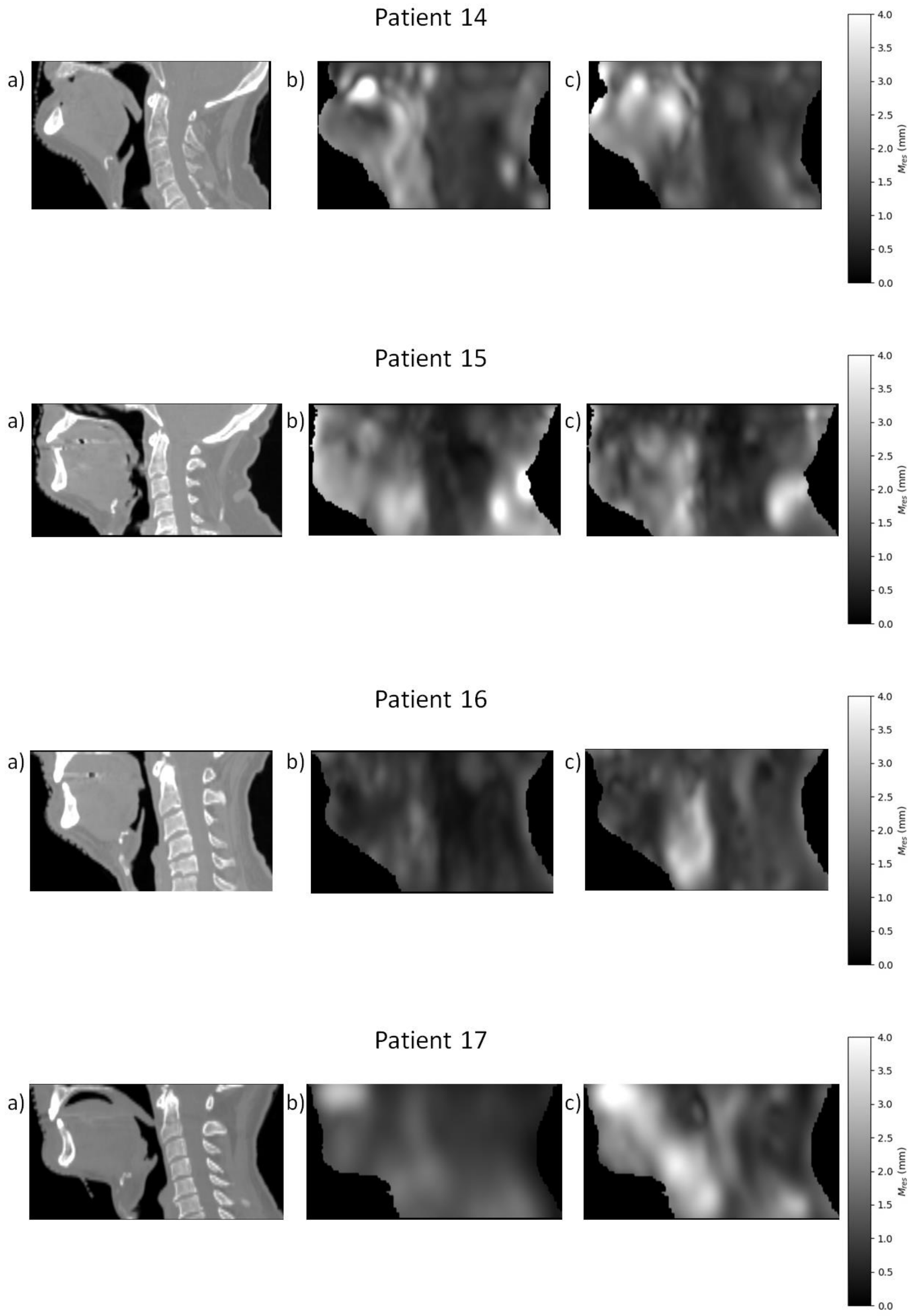

Patient 14
a)
b)
c)
4.0
3.5
3.0
2.5
2.0
1.5
1.0
0.5
0.0
M_{res} (mm)
Patient 15
a)
b)
c)
4.0
3.5
3.0
2.5
2.0
1.5
1.0
0.5
0.0
M_{res} (mm)
Patient 16
a)
b)
c)
4.0
3.5
3.0
2.5
2.0
1.5
1.0
0.5
0.0
M_{res} (mm)
Patient 17
a)
b)
c)
4.0
3.5
3.0
2.5
2.0
1.5
1.0
0.5
0.0
M_{res} (mm)

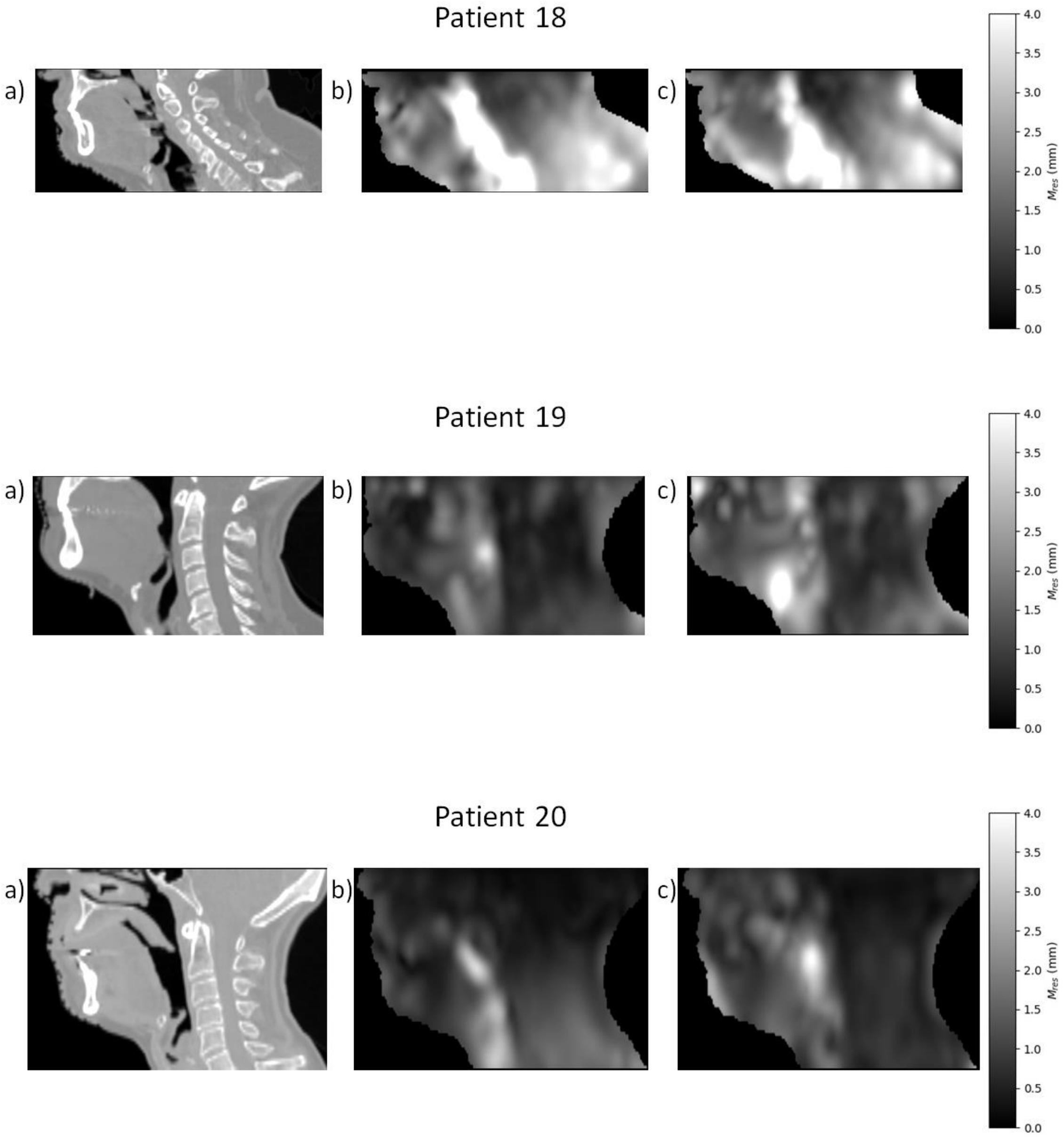
Patient 18
a)
b)
c)
Patient 19
a)
b)
c)
Patient 20
a)
b)
c)
4.0
3.5
3.0
2.5
2.0
1.5
1.0
0.5
0.0
M_{res} (mm)

Supplement 7

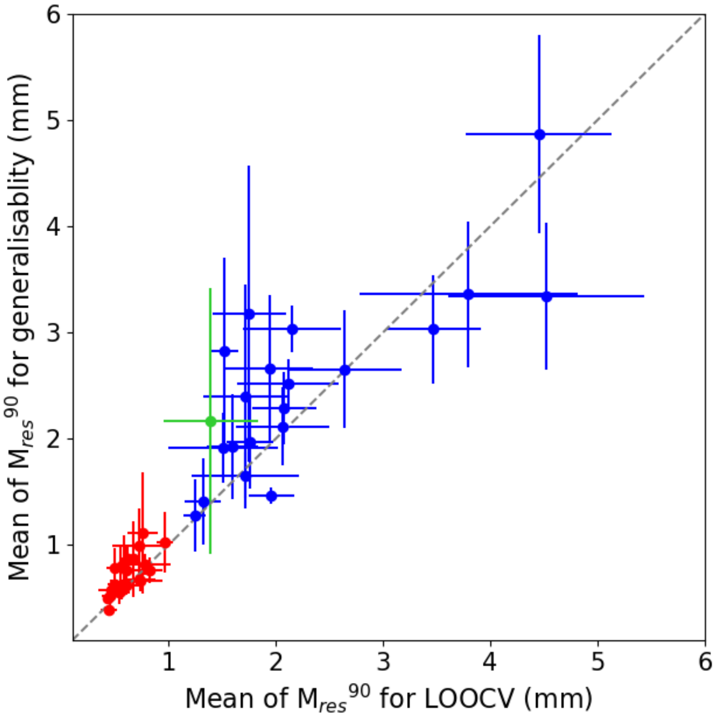

Supplementary Figure S9. Scatter plot of the mean $M_{res}{}^{90}$ for LOOCV (with bars extending +/- 1SD) with the mean $M_{res}{}^{90}$ for generalisability (with bars extending +/- 1SD) for dataset 1 (red), dataset 2 (blue) and dataset 3a (green).

Supplement 8

For the population-based model, the validation patients performed worse in the evaluation than the training patients, likely due to systematic differences between the patients. For example, the pCT of validation patient 2 (which had some of the highest $M_{res}{}^{90}$ values) shows that this patient was particularly large compared to the average pCT (see Supplementary Figure 10). This suggests that this kind of model works best on patients that are similar to the average patient geometry.

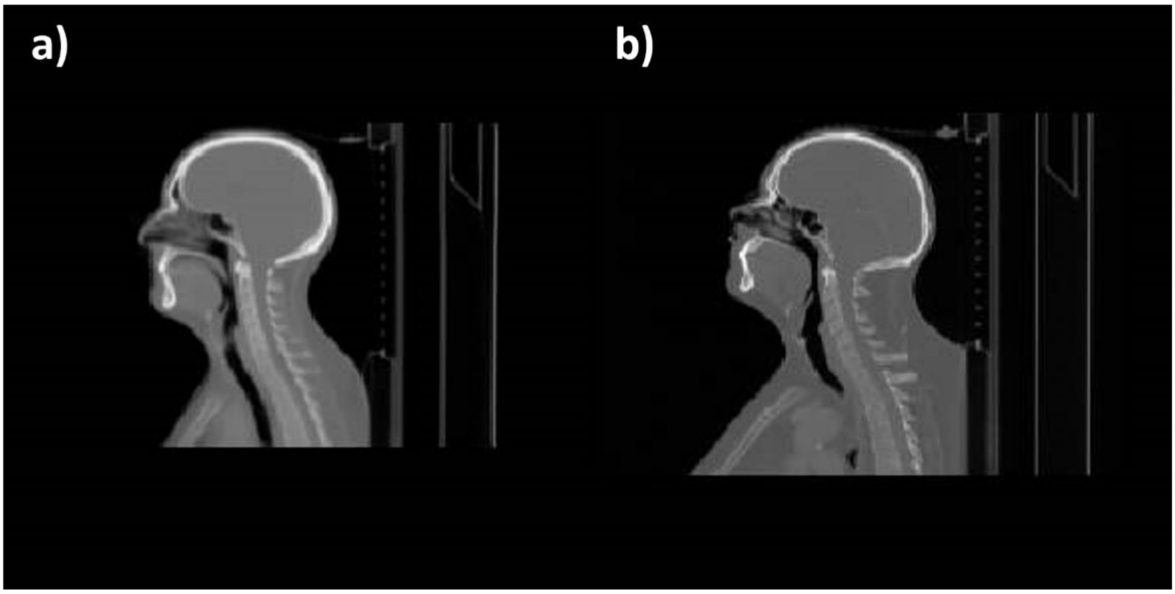

Supplementary Figure S10. a) the average pCT of the training patients and b) the pCT of validation patient 2 which showed consistently high $M_{res}{}^{90}$ values for all scans evaluated against.
